# Process Modeling and Convective Drying Optimization of Raspberry Pomace as a Fiber-Rich Functional Ingredient: Effect on Techno-Functional and Bioactive Properties

**DOI:** 10.3390/foods13223597

**Published:** 2024-11-11

**Authors:** José P. Tejeda-Miramontes, Brenda C. Espinoza-Paredes, Ana Zatarain-Palffy, Tomás García-Cayuela, Viridiana Tejada-Ortigoza, Luis Eduardo Garcia-Amezquita

**Affiliations:** 1 Tecnologico de Monterrey, Escuela de Ingenieria y Ciencias, Campus Guadalajara, Ave. General Ramón Corona 2514, Zapopan 45138, Mexico; jp.tejedamx@outlook.com (J.P.T.-M.); brendacristinaespinoza@outlook.com (B.C.E.-P.); zatarainana@gmail.com (A.Z.-P.); tomasgc@tec.mx (T.G.-C.); 2 Tecnologico de Monterrey, Escuela de Ingenieria y Ciencias, Campus Monterrey, Ave. Eugenio Garza Sada 2501, Monterrey 64849, Mexico; viri.tejada@tec.mx

**Keywords:** raspberry pomace, convective drying, drying kinetics, mathematical modeling, dietary fiber, techno-functional properties, antioxidant activity

## Abstract

This study aimed to transform raspberry pomace, a by-product of the berry industry, into a sustainable, fiber-rich functional ingredient using convective drying. Drying experiments were conducted at temperatures of 50, 60, 70, 80, and 90 °C to identify the optimal conditions that balance process efficiency and preservation of functional and bioactive properties. The best results were achieved at 70 °C, where a high drying rate (*DR*) of 0.46 kg H_2_O·kg^−1^ db·min^−1^, effective moisture diffusivity (*D*_eff_) of 1.53 × 10^−10^ m^2^·s^−1^, and activation energy (*E*_a_) of 34.90 kJ·mol^−1^ were observed. The Page model accurately represented the drying behavior (*R*^2^ = 0.9965−0.9997). Total dietary fiber (TDF) content remained stable across temperatures (52.52–64.76 g·100 g^−1^ db), while soluble dietary fiber (SDF) increased by 43.40%, resulting in a solubility (SOL) of 71.8%, water-holding capacity (WHC) of 8.2 mL·g^−1^ db, and oil-holding capacity (OHC) of 3.0 mL·g^−1^ db. High retention of bioactive compounds was achieved at 70 °C, including phenolics (32.10 mg GAE·g^−1^ db) and anthocyanins (25.84 mg C3G·g^−1^ db), resulting in significant antioxidant activities (DPPH: 33.29 mg AAE·g^−1^ db, IC_50_ 0.016 mg·mL^−1^; ABTS: 35.85 mg AAE·g^−1^ db, IC_50_ 0.029 mg·mL^−1^). These findings demonstrated the potential of convective drying at 70 °C to efficiently transform raspberry pomace into a high-quality functional ingredient. This process promotes sustainable production and waste reduction in the berry industry.

## 1. Introduction

Food waste is a pressing global concern, contributing to significant environmental and economic impacts, including the annual emission of 4.4 billion tons of greenhouse gases [1]. A considerable portion of this waste stems from fruit by-products, such as pomace, which is the solid residue remaining after juice or oil extraction from fruits. Although often discarded, pomace represents an opportunity for sustainable reuse and resource optimization [2]. In particular, raspberry pomace has attracted interest due to its rich composition of dietary fiber and bioactive compounds, including phenolic compounds and anthocyanins, which are well-known for their antioxidant and anti-inflammatory properties [3,4,5]. Despite its nutritional potential, large quantities of raspberry pomace remain underutilized, exacerbating environmental challenges [6]. Transforming this nutrient-rich by-product into a functional food ingredient could reduce waste while enhancing food sustainability [7]. However, developing effective strategies that include methods such as optimized drying techniques to preserve its bioactive and techno-functional properties during processing is essential to unlocking its full potential [4,8] and ensuring that it meets the quality standards for food applications [9].

Various drying techniques have been explored to stabilize the fruit pomace and extend its shelf life [10,11]. Freeze-drying and vacuum drying are particularly effective in preserving product quality; however, their high energy consumption and prolonged processing times present significant drawbacks [9]. Alternative methods, such as microwave and infrared drying, offer faster moisture removal but require precise control to avoid uneven heating and potential quality degradation [12,13,14]. Convective drying is a widely utilized method in the food industry, balancing moisture removal with the retention of functional properties and enabling larger sample volumes to be processed efficiently [9,14,15,16]. Nevertheless, the high fiber content and porous nature of raspberry pomace pose challenges, including uneven drying and potential quality degradation if the process is not properly optimized [4]. Therefore, achieving optimal convective drying conditions is critical for maintaining bioactive compounds and enhancing dietary fiber and techno-functional properties, thereby facilitating their successful application in various food formulations [5,17].

Optimizing the drying process of raspberry pomace requires a thorough understanding of its drying kinetics. Mathematical modeling has proven valuable for evaluating and predicting the drying behavior of various food by-products, enabling process adjustments that help preserve bioactive compounds and functional properties [16,18,19,20]. Applying such models to the convective drying of raspberry pomace can provide insights into the influence of specific drying conditions on moisture loss and quality retention, addressing existing gaps in the literature [19,20]. These models do more than predict drying curves; they facilitate the design of scalable and energy-efficient industrial processes, allowing manufacturers to balance efficiency and product quality. Given the dense fibrous structure and unique bioactive composition of raspberry pomace, the development of precise and tailored drying models is essential for achieving high-quality results. Such modeling supports energy-efficient practices, minimizes processing time, and ensures the retention of critical properties that enhance the value of raspberry pomace as a functional food ingredient [17], addressing both economic and environmental concerns in the food industry.

The drying process can significantly impact the quality of raspberry pomace, influencing its dietary fiber composition, techno-functional properties, and bioactive compound retention. Thermal treatments, such as those used in drying, can alter the structure of dietary fiber, potentially modifying its solubility and digestibility through processes such as depolymerization [21,22]. However, such treatments may also degrade heat-sensitive bioactive compounds, including phenolic compounds and anthocyanins, which contribute to the antioxidant activity [12,23]. Although research has examined the effects of drying on pomaces from other fruits, such as grapes, cranberries, blueberries, and apples, these findings vary depending on the composition and processing conditions. The distinct composition of raspberry pomace necessitates targeted research to understand how the drying conditions affect its properties [5,19,24]. Exploring the impact of different drying temperatures on fiber composition, techno-functional attributes (such as water- and oil-holding capacity), and retention of bioactive compounds in raspberry pomace can provide essential insights into preserving its nutritional and functional qualities, thus expanding its potential applications in the food industry.

This study aimed to bridge the knowledge gap by integrating mathematical modeling and experimental validation to identify the optimal convective drying temperature for raspberry pomace, transforming it into a fiber-rich, functional ingredient. A temperature range of 50–90 °C was selected to encompass moderate to high industrial drying conditions. Although higher temperatures, such as 90 °C, may reduce bioactive compounds, they can enhance dietary fiber solubility and techno-functional attributes [9]. Including 90 °C in the study enables assessment of whether the benefits of improved fiber properties and increased drying efficiency can offset potential reductions in bioactive compounds, providing a comprehensive understanding of optimal drying conditions. By comparing commonly used semi-empirical drying models, this study provides detailed insights into the drying kinetics of raspberry pomace, contributing to the development of efficient drying protocols that balance process efficiency and quality preservation. These findings support the sustainable valorization of raspberry pomace, promoting waste reduction and enhancing its role as a high-value ingredient in food production, aligning with the broader objectives of sustainability and resource optimization within the food industry.

## 2. Materials and Methods

### 2.1. Material and Preparation

All reagents and chemicals used for analysis were of analytical grade. The Folin-Ciocalteu reagent, 2,2-diphenyl-1-picrylhydrazyl (DPPH), 2,2′-azino-bis (3-ethylbenzothiazoline)-6-sulfonic acid (ABTS), gallic acid, ascorbic acid, potassium persulfate, and cyanidin-3-glucoside standard were purchased from Sigma-Aldrich (Saint Louis, MO, USA). HPLC-grade methanol and all reagents used for protein and fat determination were obtained from CTR Scientific (Zapopan, Jalisco, Mexico). Enzymatic kits for dietary fiber analysis (K-INTDF 06/13) were acquired from Megazyme (Wicklow, Ireland).

Raspberries (*Rubus idaeus*, var. ‘Adelita’) of second and third quality were acquired from local markets in Guadalajara, Mexico. The selection criteria were uniform size, color, and absence of visible damage. The samples were subsequently transported to the facilities of Tecnológico de Monterrey for analysis. The initial quality assessment involved measuring various physicochemical properties, such as pH, °Brix, titratable acidity, maturity index, and color (L*, a*, b*) as shown in Table S1, following AOAC methods 981.12, 932.12, and 942.15 [25,26,27].

The raspberries were washed, dried with absorbent paper, and pressed to extract juice using a manual citrus press (Vevor, YZQ-6A, Shanghai, China), yielding raspberry pomace. The pomace obtained was immediately homogenized using a food processor (IKA-Werke GmbH & Co. KG, A10 basic, Staufen, Germany) to achieve a uniform particle size. The homogenized pomace was then stored at 4 °C in airtight containers and processed within 24 h to minimize any degradation of bioactive compounds.

### 2.2. Experimental Set-Up and Drying Procedure

A dryer (Labotech, BDI-51, Mexico City, Mexico) was used to dry raspberry pomace (0.250 ± 0.001 kg per batch). The pomace was placed in a 0.25 × 0.20 × 0.01 m tray to ensure a uniform thickness of 0.01 m, verified with a caliper (Mitutoyo, CD-6” CSX, Kawasaki, Japan), and positioned in an aluminum container. Drying was performed at 50, 60, 70, 80, and 90 °C with a constant airflow of 2.5 m∙s^−1^. These temperatures were selected to represent a range of moderate to high drying conditions commonly used in industrial convective drying processes [9,12]. A constant airflow rate was maintained to ensure uniform drying conditions across all the samples. Moisture loss was recorded at 30-min intervals using an analytical balance (precision ±0.001 g) until the change in weight was less than 0.01% over three consecutive measurements, indicating that equilibrium moisture content had been reached.

Each drying condition was repeated three times to ensure consistency. A completely randomized design was used with three replicates for each drying temperature to assess the effect of temperature on the drying kinetics and product quality. After drying, the pomace was crushed using the IKA food processor and sieved through a 40-mesh screen (0.425 μm). The dried pomace powder was stored in vacuum-sealed low-density polyethylene bags, shielded from light for subsequent analyses. Bioactive compounds were evaluated after one day and techno-functional properties within 15 days.

### 2.3. Drying Kinetics

The moisture content (kg H_2_O∙kg^−1^ dry basis (db)) of the raspberry pomace was monitored throughout the drying process and expressed as the moisture ratio (*MR*, dimensionless) using Equation (1):

(1) $$MR=\frac{M_{t}-M_{e}}{M_{0}-M_{e}}$$

where *M*_t_ represents the moisture content at time *t* (min); *M*_0_ is the initial moisture content; and *M*_e_ is the equilibrium moisture content (kg H_2_O∙kg^−1^ db), which is considered to be reached when the change in weight is less than 0.01% over three consecutive measurements [17]. Moisture content was assessed using the weight difference approach, with weights measured using an analytical balance (precision ± 0.001 g).

Drying rate (*DR*, kg H_2_O∙kg^−1^ db∙min^−1^) was calculated every 30 min using Equation (2):

(2) $$DR=\frac{M_{t_{1}}-M_{t_{2}}}{t_{1}-t_{2}}$$

where *M*_t1_ and *M*_t2_ are the moisture contents at times *t*_1_ and *t*_2_, respectively [16].

Scanning electron microscopy (SEM) was used to examine the microstructural effects of drying temperature on raspberry pomace. The SEM methodology was adapted from Li et al. [4], with modifications to suit the current study. The samples were mounted on aluminum stubs with carbon adhesive tape and sputter-coated with a thin layer of gold to improve conductivity and prevent charging artifacts. Imaging was conducted using a scanning electron microscope (Phenom-World, Phenom ProX, Eindhoven, The Netherlands) operating at an accelerating voltage of 10 kV and a magnification of 1000×. Micrographs were obtained for samples dried at 50, 70, and 90 °C, representing the low, intermediate, and high ends of the temperature range studied. These specific temperatures were selected to observe the progression of structural changes associated with surface hardening across the drying spectrum.

### 2.4. Drying Process Modeling

Experimental data from the drying of raspberry pomace were fitted to five mathematical models to characterize thin-layer drying kinetics at various temperatures (Table 1). The selected models, Page, Modified Page, Henderson and Pabis, Logarithmic, and Midilli, are widely recognized for their applicability in modeling the drying behavior of agricultural products due to their capacity to accurately describe moisture loss patterns under drying conditions [16,18,19,20]. These models have been successfully applied in previous studies involving the drying kinetics of fruit and pomace materials, which supports their selection for assessing raspberry pomace drying behavior.

Model parameters (*a*, *b*, *c*, *k*, and *n*) were estimated using non-linear regression through the Solver function in Microsoft Excel (version 2306), adjusting parameters to minimize the sum of squared errors between the experimental and predicted *MR* values.

The goodness of fit for each model was evaluated using statistical criteria, including the coefficient of determination (*R*^2^), chi-square (*χ*^2^), mean squared error (*MSE*), sum of squared errors (*SSE*), root mean square error (*RMSE*), and corrected Akaike information criterion (*AIC*) (Table 2). These evaluations were used to identify the model that most accurately represented drying kinetics, facilitating the extrapolation of laboratory results to industrial applications.

### 2.5. Effective Moisture Diffusivity and Activation Energy

The effective moisture diffusivity (*D*_eff_, m^2^∙s^−1^) was determined to analyze the drying kinetics of the raspberry pomace. Fick’s second law of diffusion for one-dimensional moisture transport in a slab was initially applied, as shown in Equation (3).

(3) $$MR=\frac{8}{\pi^{2}}exp\left(\frac{-\pi^{2}D_{cal}}{4L^{2}}t\right)$$

where *D*_cal_ is the calculated moisture diffusivity (m^2^∙s^−1^), *L* is the half-thickness of the sample (m), and *t* is the drying time (min) [17].

Applying the natural logarithm to both sides of Equation (3) gives:

(4) $$\ln\left(MR\right)=ln\left(\frac{8}{\pi^{2}}\right)-\frac{-\pi^{2}D_{cal}}{4L^{2}}t$$

The values of *D*_cal_ were obtained from the slope of the linear regression of ln(*MR*) versus the drying time *t* (Figure S1). However, since Fick’s model assumes constant diffusivity and does not account for acceleration phases or surface changes during drying, the Weibull model was employed for a more accurate representation of drying kinetics [30].

To calculate *D*_eff_, *D*_cal_ was adjusted using the geometric factor *R*_g_ as shown in Equation (5):

(5) $$D_{eff}=\frac{D_{cal}}{R_{g}}$$

where *R*_g_ is a dimensionless geometric factor (13.1) specific for flat surfaces in the pomace sample geometry.

The activation energy (*E*_a_, kJ∙mol^−1^) was calculated by analyzing the relationship between *D*_eff_ and temperature using the Arrhenius equation (Equation (6)):

(6) $$D_{eff}=D_{0}exp\left(-\frac{E_{a}}{R\left(T+273.15\right)}\right)$$

where *D*_0_ is the pre-exponential factor (m^2^∙s^−1^), *R* represents the universal gas constant (8.314 kJ∙mol^−1^·K^−1^), and *T* denotes the temperature (°C) [28]. The values of *D*_0_ and *E*_a_ were derived through non-linear regression of ln(*D*_eff_) versus the inverse temperature (1∙T^−1^, K) using Microsoft Excel (Figure S2).

### 2.6. Quality and Functionality of DRP After Convective Drying

The influence of convective drying on the quality of dried raspberry pomace (DRP) was assessed by examining changes in the distribution of dietary fiber fractions (both insoluble and soluble). These fractions influence the functional properties of the final product [9], including the techno-functional characteristics and bioactive compound content. Freeze-drying was selected as the reference method due to its well-documented effectiveness in preserving both nutritional and bioactive compounds [23]. The process was performed at –88 °C and 0.045 mbar using a freeze-drying unit (Labconco, FreeZone 4.5, Kansas City, MO, USA).

#### 2.6.1. Dietary Fiber Composition

Total dietary fiber (TDF), including insoluble (IDF) and soluble (SDF) fractions, was determined in DRP using a modified AOAC 2011.25 method described by Garcia-Amezquita et al. [21] and the Megazyme Total Dietary Fiber Assay Kit (KTDFR, Wicklow, Ireland). Briefly, one gram of DRP was combined with 1 mL of 96% ethanol and 40 mL of maleate buffer containing pancreatic α-amylase (50 U∙mL^−1^) and amyloglucosidase (3.4 U∙mL^−1^). The mixture was incubated at 37 °C for 16 h with shaking at 150 rpm to facilitate enzymatic activity. The reaction was stopped by adding 3.0 mL of 0.75 M Tris, and the flasks were heated in a water bath at 95 °C for 20 min. After cooling to 60 °C, 0.1 mL of protease solution (350 U∙mL^−1^ tyrosine) was added, and the mixture was incubated at 60 °C for 30 min. The pH was adjusted to 4.3 with 4.0 mL of 2 M acetic acid.

Samples were filtered through fritted crucibles containing 2 g of Celite^®^, and the retained fraction was dried at 110 °C overnight to determine the IDF residue. The filtrate was combined with 280 mL of 95% ethanol preheated to 60 °C and filtered again through fritted crucibles containing 2 g Celite^®^ to isolate the SDF residue. The IDF and SDF fractions were corrected by measuring and subtracting the residual protein and ash content from the final weight. TDF was calculated as the sum of IDF and SDF (g∙100 g^−1^ db).

#### 2.6.2. Techno-Functional Properties

The techno-functional properties of DRP were analyzed to assess its potential applications in food products, following the method by Garcia-Amezquita et al. [21]. The properties evaluated included solubility (SOL, %), water-holding capacity (WHC, mL∙g^−1^), oil-holding capacity (OHC, mL∙g^−1^), swelling capacity (SC, mL∙g^−1^), and tapped density (TD, kg∙m^−3^). Briefly, SOL was assessed by dispersing 200 mg of DRP in 30 mL of water, stirring for 3 h at 25 °C, and centrifuging at 3000× *g* for 25 min at 15 °C. The residue was washed, filtered, dried at 60 °C for 24 h, and then weighed. WHC and OHC were determined by mixing 500 mg of DRP with 10 mL of water or oil, respectively, vortexing for 5 min, and allowing the mixture to rest for 18 h. The samples were centrifuged at 4500× *g* and 22 °C for 30 min. For WHC, the pellets were weighed before and after oven-drying at 60 °C. OHC was determined from the final volume after centrifugation. SC was measured by suspending 200 mg of DRP in 10 mL of water, stirring for 2 min, and letting it rest at 25 °C for 24 h; initial and final volumes were recorded. TD was assessed by filling a 10 mL test tube with the sample, tapping it manually 500 times to pack it uniformly, and recording the final volume. All measurements were performed in triplicate to ensure accuracy.

#### 2.6.3. Bioactive and Antioxidant Properties

Bioactive compounds and antioxidant activity of DRP were determined to assess the impact of convective drying. Methanolic extracts of DRP were prepared for the analysis of total phenolic content (TPC, mg GAE·g^−1^ db), total anthocyanin content (TAC, mg C3G·g^−1^ db), and antioxidant capacity (mg AAE·g^−1^ db) using DPPH and ABTS assays. Briefly, TPC was measured using the Folin–Ciocalteu method with absorbance at 765 nm and gallic acid as the standard (5–200 μg∙mL^−1^) [3]. TAC was determined by the pH differential method, with absorbance readings at 510 and 700 nm, using cyanidin-3-glucoside as the standard [23]. Antioxidant capacity was assessed by ABTS and DPPH assays as described by İzli et al. and Gouw et al., with absorbance measured at 734 nm for ABTS and 515 nm for DPPH, using ascorbic acid as the standard (5–130 μg∙mL^−1^) [3,29].

Serial dilutions of the methanolic extracts were prepared within the concentration range specified for the standards to calculate the IC_50_ values [10]. The percentage inhibition (% inhibition) was determined according to Equation (7), and IC_50_ values were derived through non-linear regression analysis using Microsoft Excel.

(7) $$\%\;Inhibition=\left(1-\frac{A_{c}-A_{s}}{A_{c}}\right)\times 100\;$$

where *A*_c_ denotes the absorbance of the control, and *A*_s_ represents the absorbance of the sample.

### 2.7. Statistical Analysis

Results are presented as mean ± standard deviation (SD), with experiments performed in triplicate. Statistical analyses were performed using Minitab 17. An analysis of variance (ANOVA), followed by Tukey’s multiple comparison test, was conducted to determine significant differences among drying temperatures (50–90 °C) at α = 0.05. Data normality and homogeneity of variances were assessed using the Shapiro–Wilk and Levene’s tests, respectively. All statistical tests were two-tailed, and a *p*-value below 0.05 was considered significant.

## 3. Results and Discussion

### 3.1. Drying Kinetics

**Figure 1:**
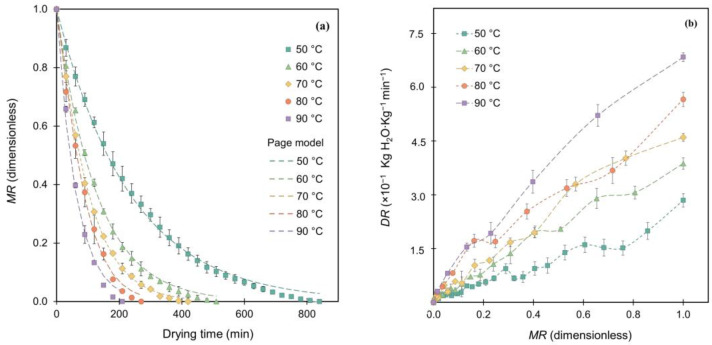
Drying kinetics of raspberry pomace: (**a**) time-dependent changes in moisture ratio (*MR*) at varying temperatures (50, 60, 70, 80, and 90 °C), fitted to the Page model; (**b**) drying rate (*DR*) as a function of *MR* at corresponding temperatures for raspberry pomace.

Figure 1 illustrates the effect of different drying temperatures (50–90 °C) on moisture reduction in raspberry pomace during convective drying. An increase in temperature led to a significant decrease in *MR* (Figure 1a), shortening the drying time from 810 to 210 min—a 74% reduction. This reduction implies potential energy savings and enhanced production capacity, both of which are beneficial for industrial applications. Figure 1b indicates an increase in the *DR* from 2.84 to 6.83 × 10^−1^ kg H_2_O∙kg^−1^ db∙min^−1^ as temperature rose, directly contributing to the reduced drying time. This can be attributed to the higher kinetic energy of water molecules, which increase molecular vibrations, generate internal heat, and raise vapor pressure within the material. The increased energy likely enables the molecules to overcome intermolecular forces, facilitating more efficient evaporation [31]. However, while higher temperatures improve drying efficiency, they may also degrade or alter the functional and bioactive properties of the product. Therefore, optimizing the drying conditions requires balancing energy savings with preserving the quality of the final product.

Mierzwa et al. [15] reported a drying time of 930 min (55 °C, 0.4 m·s^−1^, 19 mm) with an *MR* of 9.90 × 10^−3^ in a previous study on raspberry drying, compared to the 810 min observed at 50 °C in this work. These differences could be due to the smaller sample thickness (10 mm) and higher airflow rate (2.5 m·s^−1^) used in this study, which likely reduced moisture transport resistance and enhanced mass transfer rates [32]. Szadzińska et al. [33] reported a *DR* of 2.40 × 10^−3^ kg H_2_O∙kg^−1^ db∙min^−1^ (55 °C, 2.0 m·s^−1^, 23 mm), while Mierzwa et al. [15] documented 4.10 × 10^−3^ kg H_2_O∙kg^−1^ db∙min^−1^ (55 °C, 2.0 m·s^−1^, 19 mm). In this study, a significantly higher *DR* of 5.90 × 10^−1^ kg H_2_O∙kg^−1^ db∙min^−1^ (50 °C, 2.5 m·s^−1^, 10 mm) was observed, suggesting that optimizing the airflow and sample thickness could improve drying performance compared with previous conditions.

**Figure 2:**
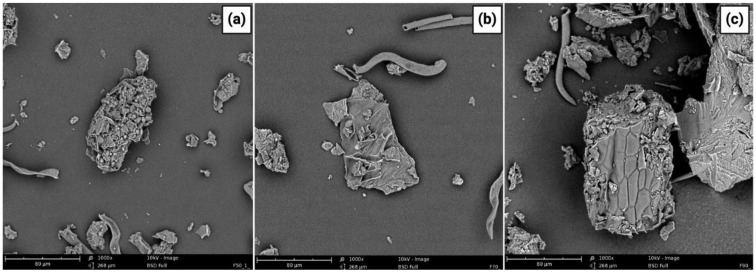
SEM micrographs of raspberry pomace at drying treatments: (**a**) 50 °C, (**b**) 70 °C, and (**c**) 90 °C.

The equilibrium *MR* increased from 3.50 × 10^−3^ to 1.54 × 10^−2^ as drying temperatures rose, indicating higher moisture retention in the final product. Previous studies have demonstrated that increases in drying temperature (50 to 60 °C) can induce surface hardening in jackfruit, restricting moisture transfer from the interior to the surface and thereby increasing moisture retention within the product [34]. SEM analysis of raspberry pomace dried at 50, 70, and 90 °C, representing low, intermediate, and high temperatures, respectively (Figure 2), provided insights into these microstructural changes.

At 50 °C (Figure 2a), the pomace exhibited a loosely packed structure with minimal cell collapse, suggesting that low-temperature drying may help preserve the cell wall integrity and facilitate efficient moisture release. At 70 °C (Figure 2b), partial cell collapse and densification were observed, indicating the initial stages of surface hardening. At 90 °C (Figure 2c), the SEM micrograph showed significant structural compaction, consistent with severe surface hardening. This progression in microstructural changes with increasing temperature may explain the observed increase in moisture retention by limiting moisture release from the inner matrix to the surface. These findings are consistent with those reported by Leelawat et al. [34], who observed a similar relationship between surface hardening and increased moisture retention in jackfruit. Confocal laser scanning microscopy could be explored in future studies to provide complementary insights into microstructural changes, offering a more detailed view of cell wall integrity and moisture distribution under varying drying conditions.

In industrial applications, surface hardening must be carefully controlled, as this phenomenon can compromise product texture and reduce shelf life by restricting moisture migration from the interior to the surface. Retained moisture may increase internal water activity, raising the risk of microbial growth and compromising storage stability. Even low-moisture foods can retain viable pathogens during storage, highlighting the critical importance of effective moisture control and careful optimization of drying conditions to ensure both product safety and quality [35]. Optimizing drying conditions to minimize surface hardening and preserve texture is essential for extending the shelf life of raspberry pomace while balancing energy efficiency and high product quality in industrial applications.

### 3.2. Drying Process Modeling

Five mathematical models were employed to study the drying kinetics of raspberry pomace, as shown in Table 1, to characterize and predict drying behavior. Table 3 summarizes the parameters obtained from each model and the corresponding statistical metrics, offering insights into key aspects of the drying process.

Table 3 shows that the constant *k*, which represents the *DR* constant, increased with temperature across all models, highlighting the role of temperature in accelerating the drying process. The highest *k* values were recorded in the Modified Page model (0.5064–0.9867), followed by the Page model (0.3344–0.9282). The elevated *k* values in the Modified Page model suggest that this model may be more suitable for processes prioritizing drying speed over product quality [20]. The curvature factor *n* also increased with temperature, which may indicate shifts in *DR* dynamics. Higher *n* values (>1) in the Page model (1.0569 to 1.1896) suggest a faster initial *DR*, which could be beneficial for rapidly reducing moisture and thereby limiting microbial growth and spoilage [36]. Conversely, lower *n* values (<1) in the Modified Page model (0.5063 to 0.9867) suggest a more gradual *DR*, which may help preserve product quality by preventing over-drying [9,13].

The constant *a*, associated with the initial moisture content of the samples, showed similar values in the Midilli model (1.0134–1.0220) and the Henderson–Pabis and Logarithmic models (1.0082–1.0242), which may suggest consistent initial moisture conditions across varying temperatures. This consistency is crucial for repeatability and enhances the reliability of these models in process standardization [13]. The absence of constants *b* and *c* in the Midilli and Logarithmic models suggests that fewer parameters are needed to describe the drying kinetics, potentially simplifying the modeling process [9]. This simplification may be advantageous in practical applications where computational efficiency is essential, as it reduces model complexity.

Statistical analysis showed that all the models provided a strong fit to the drying kinetics, with *R*^2^ values ranging from 0.9867 to 0.9997. However, among the models, the Page model demonstrated the best fit (Table 3), with higher *R*^2^ values (0.9965–0.9997) and lower values for *χ*^2^ (0.0242–0.0849), *MSE* (0.0002–0.0004), *RMSE* (0.0046–0.0201), *SSE* (0.0006–0.0041), and *AIC*_C_ (−249.5219 to −66.5573) across all temperatures. As illustrated in Figure 1a, the experimental and predicted *MR* values using the Page model at various temperatures demonstrate the excellent fit of this model to the data. This performance aligns with its reported effectiveness for other berries, such as blueberries and cranberries, where it has demonstrated high accuracy under varying conditions [19,28]. The ability of this model to capture both the initial drying rate (*n*) and the curvature factor (*k*) may support effective moisture management, establishing it as a reliable tool for predicting drying kinetics in raspberry pomace. Overall, the Page model appears to be a valuable tool for optimizing industrial drying processes, aiding in the maintenance of product quality.

### 3.3. Effective Moisture Diffusivity and Activation Energy

**Figure 3:**
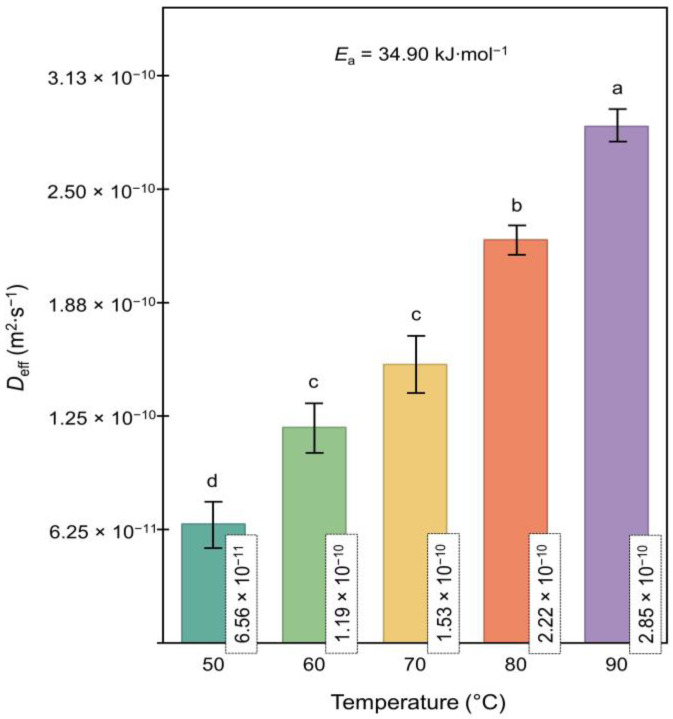
Effective moisture diffusivity (*D*_eff_) of raspberry pomace at different drying temperatures (50, 60, 70, 80, and 90 °C). The activation energy (*E*_a_) was calculated as 34.90 kJ·mol^−1^. Significant differences between treatments (*p* < 0.05) are indicated by different letters above the bars.

As shown in Figure 3, *D*_eff_ increased significantly from 6.56 × 10^−11^ to 2.85 × 10^−10^ m^2^∙s^−1^ as the drying temperature rose from 50 to 90 °C (*p* < 0.05), indicating enhanced moisture diffusion at higher temperatures. This 335% increase highlights the substantial impact of elevated drying temperatures on moisture diffusion in raspberry pomace, aligning with findings for other fruits such as blueberries and mangoes, where higher temperatures similarly enhanced diffusivity [16,28]. The *E*_a_ of 34.90 kJ∙mol^−1^, calculated using the Arrhenius model, indicates the energy required to overcome the moisture diffusion barriers in the raspberry pomace. This value is notably higher than those reported for blueberry pomace (23.12 kJ∙mol^−1^) and grape pomace (27.56 kJ∙mol^−1^) [18,28], suggesting greater resistance to moisture diffusion in raspberry pomace.

This higher *E*_a_ value compared to other fruit pomaces may be attributed to the unique microstructural composition of raspberry pomace, which includes substantial amounts of insoluble fiber and phenolic compounds that could restrict water movement within the matrix [5,37]. Elevated temperatures can reduce structural barriers such as cell walls and intercellular spaces. However, the drying process can also induce significant interactions between macromolecules, such as polysaccharides, proteins, and polyphenols, potentially leading to the formation of complexes that contribute to matrix densification. As demonstrated in previous studies, these interactions, often facilitated by elevated temperatures, can result in a denser structure, which further restricts moisture diffusion and may influence product stability due to localized areas of higher water activity [38,39].

Understanding these factors is essential for optimizing the drying process to achieve effective moisture removal while preserving the nutritional and functional properties of raspberry pomace. The findings from this study provide a basis for optimizing future drying conditions to maximize *D*_eff_ while maintaining product quality. Adjusting drying temperatures to enhance moisture diffusivity and potentially incorporating pretreatments to modify cell structure could help reduce resistance to moisture movement and lower the *E*_a_ required for diffusion. Such optimizations may improve both efficiency and product quality in industrial drying applications.

### 3.4. Quality and Functionality of DRP After Convective Drying

#### 3.4.1. Dietary Fiber Composition

Table 4 presents the TDF values from DRP, including IDF and SDF proportions, at different convective drying temperatures (50 to 90 °C). Although TDF content remained consistent across treatments (*p* > 0.05, Table 4), drying at 70 °C led to a significant shift in fiber composition. This temperature resulted in a decrease in IDF and a corresponding increase in SDF, resulting in a more favorable SDF:IDF ratio compared to the freeze-dried control (*p* < 0.05, Table 4). At 70 °C, SDF increased to 3.37 g∙100 g^−1^ db, representing a 43.40% increase over the freeze-dried control (2.35 g∙100 g^−1^ db), while IDF decreased from 61.78 to 56.03 g∙100 g^−1^ db. These results suggest that drying at 70 °C may enhance fiber solubilization by converting insoluble fibers into soluble forms. This transformation may result from the thermal degradation of polysaccharides, such as hemicellulose and cellulose, which begins with depolymerization through the cleavage of glycosidic bonds, forming smaller and more soluble carbohydrate chains at higher temperatures [40]. This thermally driven conversion process has been observed in other studies involving glycosidic bond breakdown [41,42]. The shift in the SDF:IDF ratio from 0.04:1 to 0.07:1 further supports these findings, highlighting the impact of drying temperature on the fiber composition of raspberry pomace. These insights could be valuable for optimizing processing conditions to enhance the functional properties of raspberry pomace.

The TDF values observed in this study (52.52 to 64.76 g∙100 g^−1^ db) are consistent with those reported by Fotschki et al. and Šaric et al., confirming the high fiber content in raspberry pomace [2,7]. This study specifically investigated the impact of varying drying temperatures on SDF and IDF balance, which may be significant for developing functional ingredients. Previous studies have reported IDF content at specific drying temperatures, such as 50.41 g∙100 g^−1^ db at 45 °C and 38.13 g∙100 g^−1^ db at 110 °C [3,7]. Although these studies focused on individual temperatures, their findings suggest that higher temperatures tend to reduce the IDF content. In the present study, a significant decrease in IDF was observed when the drying temperature exceeded 70 °C (*p* < 0.05), suggesting that thermal degradation may influence fiber composition. This trend is further supported by Vega-Gálvez et al. [43], who reported a decrease in IDF from 46.98 g∙100 g^−1^ db in fresh cape gooseberry to 35.45 g∙100 g^−1^ db at 80 °C, accompanied by an increase in SDF. These findings suggest that elevated drying temperatures could promote the conversion of IDF to SDF by breaking down more complex polysaccharides, thereby enhancing soluble fiber content [41,42].

Endogenous enzymes, such as pectin methyl esterase (PME) and cellulase, may also facilitate the breakdown of insoluble fiber into soluble forms, particularly at moderate drying temperatures (50–70 °C). PME catalyzes the de-esterification of pectin, enhancing its solubility, whereas cellulase breaks down cellulose into smaller, more soluble fragments [21]. Similar fiber modification has been reported in other fruit pomaces, such as apples, where thermal treatments have been shown to influence pectin solubilization and structural changes, potentially involving endogenous enzymatic activity during processing [44]. However, at higher temperatures, thermal degradation is likely to be the primary mechanism driving the increase in fiber solubility.

Variations in SDF and IDF contents may influence the potential use of DRP as a fiber-rich ingredient in food products. The significant increase in SDF content observed at 70 °C (*p* < 0.05) suggests that adjusting drying conditions could enhance fiber solubilization, consistent with studies emphasizing the importance of optimizing fiber fraction for functional applications [7]. An increase in SDF content may improve the functional properties of raspberry pomace in food applications, as SDF is associated with enhanced texture, viscosity, and potential health benefits [37]. Further investigation of enzymatic activity during drying could help clarify the mechanisms driving these fiber transformations at different temperatures. Assessing the storage stability of the modified fiber fractions is essential to determining their structural integrity over time, including evaluating whether the fibers undergo degradation or structural changes during long-term storage that could impact their performance in food formulations.

#### 3.4.2. Techno-Functional Properties

Table 5 shows the changes in the techno-functional properties (SOL, WHC, OHC, SC, and TD) of the DRP subjected to convective drying at different temperatures. At 50 °C, SOL significantly decreased to 65.2% compared with the freeze-dried control (76.0%, *p* < 0.05). This suggests that lower drying temperatures may limit solubility by restricting the breakdown of the fiber matrix, which is necessary for releasing the soluble components [22]. This observation aligns with findings in mango (50–70 °C) and pumpkin (60–80 °C), where low drying temperatures similarly limited the release of soluble components [16,29]. As the drying temperature increased, SOL reached peak values between 70 and 90 °C (71.8–72.6%), with no significant differences compared with the control (*p* > 0.05). This increase in SOL may be due to the partial hydrolysis of insoluble polysaccharides, such as hemicelluloses and cellulose, which have been shown to enhance the availability of soluble components in studies of artichoke by-products [45]. The increase in SDF observed at 70 °C (Section 3.4.1) suggests the conversion of IDF to SDF, which may contribute to the enhanced solubility at this temperature.

In contrast, studies on blueberry pomace have indicated that thermal treatments such as convective drying compared to freeze-drying can significantly reduce solubility [46]. García-Amezquita et al. [22] indicated that thermal processes can alter the dietary fiber content of different agricultural by-products, promoting interactions between hydrolyzed carbohydrates and other macromolecules, which lead to the formation of insoluble complexes. These differing results suggest that the mechanisms influencing SOL during drying may vary depending on the specific types of pomaces and plant material. Further studies are recommended to clarify the mechanisms underlying the increased SOL in DRP at higher temperatures. Enhanced solubility could make DRP suitable for applications in products such as beverages, soups, and sauces, where high solubility facilitates incorporation into liquid formulations without compromising the texture.

WHC did not show significant differences across drying temperatures from 50 to 80 °C, maintaining values at approximately 8.05 ± 0.26 mL∙g^−1^ db (*p* > 0.05). However, these values were approximately 31.78% lower than those of the freeze-dried control (11.8 mL∙g^−1^ db). This reduction may be linked to decreased porosity and structural changes observed in SEM micrographs (Figure 2), which showed a more compact microstructure at higher temperatures. Similar reductions in WHC due to decreased porosity have been reported for other fruit pomaces subjected to convective drying, such as apple pomace [24]. These findings, along with similar results for pumpkin powder (where WHC decreased from 7.5 to 6.4 mL∙g^−1^ db as drying temperature increased from 60 to 80 °C), suggest that thermal degradation frequently affects WHC in fruit materials [29]. This highlights the need to carefully regulate the drying conditions to preserve the functional properties of DRP and similar by-products. Reduced WHC at higher temperatures may limit the use of DRP in moisture-sensitive products, such as baked goods that require high moisture retention for texture and extended shelf life [47]. Therefore, maintaining drying temperatures below 80 °C may be advantageous for applications requiring higher WHC.

OHC was affected by the drying temperature. The lowest values were recorded at 50 and 60 °C (2.35 ± 0.07 mL∙g^−1^ db), representing a significant 28.79% reduction compared with the freeze-dried samples (3.3 mL∙g^−1^ db, *p* < 0.05). At 70 °C, OHC increased to 3.0 mL·g^−1^ db, similar to the control (*p* > 0.05), suggesting that this temperature may be favorable for maintaining oil retention capacity. This property is beneficial in products requiring fat absorption and emulsification, such as dressings, mayonnaise, and bakery items, where oil retention enhances mouthfeel and texture [5,48,49]. Li et al. [4] reported an OHC of 2.5 mL∙g^−1^ db at 40 °C for raspberry pomace, which aligns with our values at 50 and 60 °C, suggesting that OHC is reduced at lower drying temperatures. At 80 and 90 °C, OHC decreased to 1.7 and 2.0 mL∙g^−1^ db, respectively, significantly lower than the control (*p* < 0.05). This reduction may be attributed to the decomposition of lipophilic components, such as polyunsaturated fatty acids (linoleic and γ-linolenic acid) and tocopherols, which are mainly present in the seed fraction of the pomace [8,48]. Furthermore, structural changes in the fiber structure, including lignin and cellulose modifications, may also contribute to reducing the oil absorption [24].

SC gradually decreased with rising drying temperature, leading to a 53.95% reduction at 70–90 °C (7.0 ± 0.1 mL∙g^−1^ db) compared with the freeze-dried samples (15.2 mL∙g^−1^ db, *p* < 0.05). SEM micrographs (Figure 2) indicated that higher temperatures produced a denser, more compact microstructure, potentially restricting the ability of the fiber to absorb water and swell. Thermal processing has been shown to degrade polysaccharides in plant materials, such as Aloe vera, resulting in the breakdown of polymer chains, significant deacetylation of mannose units, and a reduction in molecular weight. These structural changes negatively affect the fibers’ ability to retain water, thereby diminishing their SC [50]. A similar effect may occur in DRP, potentially limiting its use in products where swelling is important for texture and satiety, such as cereals, snacks, and meat extenders [51]. İzli et al. [29] and Llavata et al. [24] observed similar reductions in SC in pumpkin powder (11.20−9.64 mL∙g^−1^ db, 60 to 80 °C) and apple pomace (13.18–10.61 mL∙g^−1^ db, 40 to 120 °C), aligning with these findings. Although lower temperatures (50–60 °C) resulted in higher SC, the prolonged drying times required may increase costs. Enzymatic pretreatments, as shown with green yuzu powders, can enhance SC and WHC, suggesting their use for maintaining functional properties in DRP at higher drying temperatures [52].

TD increased as the drying temperature rose. Between 50 and 80 °C, TD values ranged from 311.5 to 322.6 kg∙m^−3^ db, without significant differences (*p* > 0.05). However, at 90 °C, TD reached 362.1 kg∙m^−3^ db, indicating a significant 29% increase compared with the freeze-dried control (279 kg∙m^−3^ db, *p* < 0.05). This increase in TD may be associated with the structural compaction and reduced porosity observed in the SEM micrographs at higher temperatures (Figure 2), where cell walls appeared more compact and intercellular spaces were diminished. While a higher TD can reduce storage and transportation costs by enabling more products per unit volume, it may impact bulk density-related functionality in food applications, potentially affecting mixing properties and causing segregation or reduced mixture homogeneity [9,53]. Michalska et al. [53] observed a similar increase in plum powder density when dried between 50 and 70 °C, supporting this trend. For applications requiring lower bulk density to improve texture and volume, such as bakery products, drying at temperatures between 60 and 70 °C may help maintain a relatively low TD and preserve desirable sensory attributes.

As shown in Table 5, drying at 70 °C significantly improved the techno-functional properties of DRP, particularly SOL and OHC, while maintaining acceptable levels of WHC and SC. These findings suggest that drying at 70 °C may be optimal for enhancing these properties, indicating that DRP could serve as a functional ingredient in products with high solubility and oil retention, such as sauces, dressings, bakery goods, and meat products [48,49]. The increase in SOL and OHC at 70 °C suggests that DRP may possess additional techno-functional properties such as emulsifying, foaming, and gelling capabilities, which could broaden its applicability for improving texture in various food applications [54,55]. However, these properties were not directly evaluated in this study, highlighting a potential area for further research. The decrease in WHC and SC at higher temperatures suggests that maintaining lower drying temperatures may be crucial for applications that require moisture retention, such as bakery products. Future studies should examine the extent to which reductions in WHC and SC impact the DRP performance in such applications. Prior studies have indicated that pre-drying enzymatic treatments can help preserve porosity and specific techno-functional properties, such as water and oil retention [52]. Further research could assess the potential of these treatments to enhance the DRP performance at higher temperatures.

#### 3.4.3. Bioactive and Antioxidant Properties

**Figure 4:**
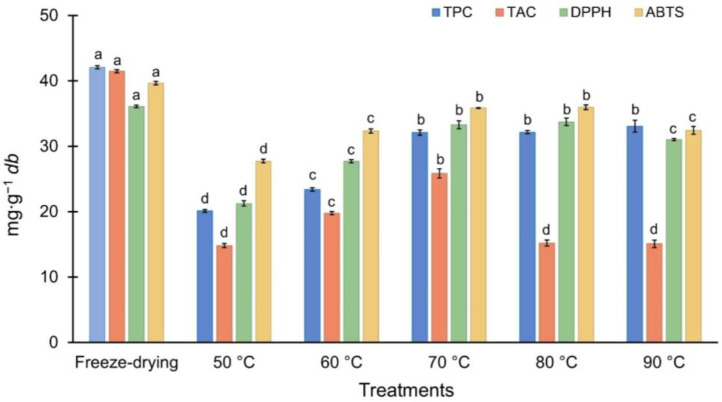
Antioxidant activity and phenolic content of DRP under different drying temperatures (50, 60, 70, 80, and 90 °C), including freeze-drying as a control. Total phenolic content (TPC, mg GAE·g^−1^ db), total anthocyanin content (TAC, mg C3G·g^−1^ db), and DPPH and ABTS activities (mg AAE·g^−1^ db). Significant differences among treatments (*p* < 0.05) are indicated by different letters (a–d) above the bars.

Figure 4 shows the TPC, TAC, and antioxidant capacity of DRP at different drying temperatures. The TPC of raspberry pomace was significantly influenced by the drying temperature. The lowest TPC was observed at 50 °C (20.13 mg GAE∙g^−1^ db, *p* < 0.05), potentially due to the prolonged drying time at lower temperatures, which increased exposure to heat and oxygen, promoting the oxidation and degradation of phenolic compounds [56]. At 70 °C, the TPC increased to 32.10 mg GAE∙g^−1^ db, significantly higher than at 50 °C (*p* < 0.05), suggesting that drying at this temperature may facilitate the release of phenolics from the pomace matrix. These findings are consistent with previous studies, like Stamenkovic et al. [23], who reported increases in TPC (1.07 to 1.28 g GAE∙100 g^−1^ db) in whole raspberries dried between 60 and 80 °C. Between 70 and 90 °C, the TPC remained stable with minor fluctuations (32.44 ± 0.55 mg GAE∙g^−1^ db), suggesting that the release of matrix-bound phenolics may offset initial thermal losses [57]. However, a 22.8% reduction compared to freeze-dried control (42.06 mg GAE∙g^−1^ db) was still observed, indicating that, while drying at these temperatures is partially effective, it does not achieve the same level of preservation as freeze-drying. Therefore, although drying at 70 °C provides a balance between processing efficiency and partial preservation of phenolic compounds, additional strategies may be needed to improve bioactive retention for the development of antioxidant-rich functional ingredients.

Anthocyanins, the compounds responsible for the vibrant red color of raspberry pomace, were particularly sensitive to drying temperature. As shown in Figure 4, at 50 °C, the TAC reached its lowest value (14.84 mg C3G∙g^−1^ db, *p* < 0.05), possibly due to prolonged exposure leading to thermal degradation, involving the breakdown of glycosidic bonds and the formation of less stable compounds [58]. The highest TAC was observed at 70 °C (25.84 mg C3G∙g^−1^ db), significantly higher than at other convective drying temperatures (*p* < 0.05), suggesting that this temperature may balance anthocyanin release and degradation. This finding implies that drying at 70 °C may be favorable for maintaining anthocyanin content in food products where natural colorants and antioxidant properties are desired [59]. However, at 80 and 90 °C, the TAC significantly decreased (15.22 and 15.10 mg C3G∙g^−1^ db, respectively, *p* < 0.05), reinforcing the notion that higher temperatures accelerate the degradation of these heat-sensitive pigments [58,60]. Despite the relative effectiveness of drying at 70 °C, there was still a 37.69% reduction compared with freeze-drying (41.47 mg C3G∙g^−1^ db), emphasizing the need to explore strategies to further protect anthocyanins during drying.

The antioxidant capacity of DRP, assessed through DPPH and ABTS assays, was also significantly influenced by drying temperature. At 50 °C, antioxidant activity was low (21.25 mg AAE∙g^−1^ db for DPPH and 27.72 mg AAE∙g^−1^ db for ABTS, *p* < 0.05), likely due to the degradation of heat-sensitive antioxidant compounds during prolonged drying [56]. This observation aligns with the reduced TPC and TAC levels observed at this temperature, suggesting similar degradation pathways for phenolic and anthocyanin compounds [46,58,60]. At 70 °C, antioxidant activity increased significantly (*p* < 0.05) to 33.29 mg AAE∙g^−1^ db for DPPH and 35.85 mg AAE∙g^−1^ db for ABTS, with corresponding IC_50_ values of 0.016 and 0.029 mg∙mL^−1^, respectively. This enhancement may result not only from better retention of antioxidant compounds but also from changes in their interactions with the pomace matrix, which can influence the overall efficacy [61]. However, at 90 °C, a decline in antioxidant activity was observed (29.14 mg AAE∙g^−1^ db for DPPH and 31.02 mg AAE∙g^−1^ db for ABTS, *p* < 0.05), and the IC_50_ values increased, indicating reduced potency at elevated temperatures. This suggests that higher temperatures accelerate the breakdown of the bioactive compounds [57]. It is important to consider that the antioxidant effectiveness of dried products may vary under different conditions, such as temperature and pH levels. These factors can influence the stability and potential autooxidation of phenolic compounds, thereby impacting their antioxidant capacity in food systems or within the human body [61]. Further studies evaluating the stability and bioactivity of these compounds under diverse conditions are necessary to fully understand their functional potential.

The observed influence of drying temperature on the retention of bioactive compounds in DRP has important implications for its application in the food and nutraceutical industries. Although drying at 70 °C appears to provide a compromise between processing efficiency and moderate preservation of phenolic compounds and anthocyanins, the significant reductions in these compounds compared to freeze-dried samples highlight the need for further optimization. Incorporating techniques that reduce the drying time and thermal exposure, such as combining microwave-assisted drying with convective drying, could be an effective solution [14]. Although microwave drying can cause uneven heating if the parameters are not precisely controlled, integrating it with convective drying could reduce the overall heat exposure, potentially minimizing thermal degradation of sensitive compounds. Optimizing this combined drying approach may enhance the bioactive retention and improve the viability of DRP as a high-value functional ingredient, supporting the development of innovative functional foods and contributing to waste reduction in the berry industry.

## 4. Conclusions

The convective drying process of raspberry pomace was successfully modeled, identifying 70 °C as the optimal temperature for producing a high-quality, fiber-rich functional ingredient. The drying kinetics were accurately described by the Page model (*R*^2^ = 0.9965–0.9997), enabling the effective prediction and optimization of the drying process. Drying at 70 °C led to significant increases in drying rate and effective moisture diffusivity, thus enhancing process efficiency. The total dietary fiber content was preserved, while soluble dietary fiber increased by 43.40%, leading to enhanced techno-functional properties such as solubility, water-holding capacity, and oil-holding capacity. In addition, high levels of phenolic compounds and anthocyanins were retained, resulting in significant antioxidant activity. Overall, these findings demonstrate that convective drying at 70 °C effectively balances process efficiency and the preservation of functional and bioactive properties, supporting the sustainable valorization of raspberry pomace in the food industry.

## Figures and Tables

**Table 1:** Mathematical models employed to characterize the drying kinetics of raspberry pomace along with their corresponding equations.

Model	Model Equation	Reference
Page	$MR=exp(-kt^{n})$	[28]
Modified Page	$MR=exp\left[(-k{t)}^{n}\right]$	[16]
Henderson and Pabis	MR=a exp (−kt)	[16]
Logarithmic	MR=a exp (−kt)+c	[9]
Midilli	$MR=a\;exp\left(-kt^{n}\right)+bt$	[9]

Model coefficients (*a*, *c*, *k*, *n*, and *b*); *t*—drying time (min).

**Table 2:** Statistical error functions employed to identify the most suitable model for describing drying kinetics.

Error Function	Reference
$R^{2}=1-\left[\frac{\sum_{i=1}^{N}{\left({MR}_{exp,i}-{MR}_{pre,i}\right)}^{2}}{\sum_{i=1}^{N}{\left({\bar{MR}}_{exp}-{MR}_{exp,i}\right)}^{2}}\right]$	[28]
$\chi^{2}=\sum_{i=1}^{N}\frac{{\left({MR}_{exp}-{MR}_{pre}\right)}^{2}}{{MR}_{pre}}$	[13]
$MSE=\frac{1}{N}\sum_{i=1}^{N}{\left({MR}_{exp,i}-{MR}_{pre,i}\right)}^{2}$	[13]
$SSE=\sum_{i=1}^{N}{\left({MR}_{exp,i}-{MR}_{pre,i}\right)}^{2}$	[23]
RMSE=1N∑i=1NMRexp,i−MRpre,i212	[29]
$AIC=N\;ln\left(\frac{SSE}{N}\right)+2K$	[9]
${AIC}_{c}=AIC+\frac{2K(K+1)}{N-K-1}$	[9]

*K* represents the number of model parameters, and *N* indicates the total number of data points used. The terms *MR*_pre_ and *MR*_exp_ refer to the predicted and experimental moisture ratios, respectively. The statistical metrics include *R*^2^ (coefficient of determination), *χ*^2^ (chi-squared statistic), *MSE* (mean squared error), *SSE* (sum of square error), *RMSE* (root mean square error), *AIC* (Akaike information criterion), and *AIC*_c_ (adjusted Akaike information criterion).

**Table 3:** Model parameters and statistical evaluation results for various thin-layer drying models.

Model	Temperature (°C)	Model Constants				*R* ^2^	*χ* ^2^	*MSE*	*SSE*	*RMSE*	*AIC*	*AIC*_c_
Page	50	*k* = 0.3344	*n* = 1.0569	-	-	0.9982	0.0849	0.0002	0.0046	0.0125	−249.9834	−249.5219
	60	*k* = 0.5325	*n* = 1.0838	-	-	0.9997	0.0242	0.0000	0.0006	0.0056	−182.4758	−181.6758
	70	*k* = 0.6635	*n* = 1.0826	-	-	0.9995	0.0277	0.0000	0.0007	0.0069	−145.1781	−144.1781
	80	*k* = 0.7454	*n* = 1.1818	-	-	0.9965	0.0539	0.0004	0.0041	0.0201	−74.1123	−72.3980
	90	*k* = 0.9282	*n* = 1.1896	-	-	0.9992	0.0276	0.0001	0.0009	0.0105	−68.9573	−66.5573
Modified Page	50	*k* = 0.5064	*n* = 0.5064	-	-	0.9981	0.1268	0.0002	0.0067	0.0152	−238.7016	−238.2400
	60	*k* = 0.6851	*n* = 0.6851	-	-	0.9992	0.0561	0.0002	0.0031	0.0132	−151.9003	−151.1003
	70	*k* = 0.7738	*n* = 0.7738	-	-	0.9991	0.0554	0.0002	0.0027	0.0134	−125.4324	−124.4324
	80	*k* = 0.8453	*n* = 0.8453	-	-	0.9938	0.1218	0.0011	0.0106	0.0326	−64.4486	−62.7343
	90	*k* = 0.9867	*n* = 0.9867	-	-	0.9963	0.0769	0.0007	0.0060	0.0273	−53.6126	−51.2126
Henderson–Pabis	50	*k* = 0.2586	-	*a* = 1.0082	-	0.9978	0.1218	0.0002	0.0066	0.0150	−239.4479	−238.9864
	60	*k* = 0.4783	-	*a* = 1.0200	-	0.9988	0.0486	0.0001	0.0025	0.0117	−156.2267	−155.4267
	70	*k* = 0.6095	-	*a* = 1.0189	-	0.9988	0.0495	0.0001	0.0022	0.0120	−128.6402	−127.6402
	80	*k* = 0.7303	-	*a* = 1.0142	-	0.9926	0.1139	0.0010	0.0099	0.0314	−65.2132	−63.4989
	90	*k* = 0.8923	-	*a* = 1.0220	-	0.9955	0.0716	0.0007	0.0054	0.0260	−54.4222	−52.0222
Logarithmic	50	*k* = 0.2586	-	*a* = 1.0082	*c* = 0.0000	0.9978	0.1218	0.0002	0.0066	0.0150	−237.4479	−236.4879
	60	*k* = 0.4783	-	*a* = 1.0200	*c* = 0.0000	0.9988	0.0486	0.0001	0.0025	0.0117	−154.2267	−152.5124
	70	*k* = 0.6095	-	*a* = 1.0189	*c* = 0.0000	0.9988	0.0495	0.0001	0.0022	0.0120	−126.6402	−124.4584
	80	*k* = 0.7303	-	*a* = 1.0142	*c* = 0.0000	0.9926	0.1139	0.0010	0.0099	0.0314	−63.2132	−59.2132
	90	*k* = 0.8923	-	*a* = 1.0220	*c* = 0.0000	0.9955	0.0716	0.0007	0.0054	0.0260	−52.4222	−46.4222
Midilli	50	*k* = 0.2748	*n* = 0.9534	*a* = 1.0200	*b* = 0.0000	0.9976	0.1890	0.0004	0.0118	0.0202	−218.4229	−216.7562
	60	*k* = 0.5522	*n* = 0.9029	*a* = 1.0134	*b* = 0.0000	0.9937	0.0908	0.0006	0.0107	0.0243	−125.7493	−122.6724
	70	*k* = 0.5643	*n* = 1.0784	*a* = 1.0190	*b* = 0.0000	0.9995	0.0287	0.0000	0.0007	0.0070	−140.9635	−136.9635
	80	*k* = 0.6296	*n* = 1.2021	*a* = 1.0169	*b* = 0.0000	0.9967	0.0514	0.0004	0.0039	0.0197	−70.5534	−62.5534
	90	*k* = 0.6296	*n* = 1.2021	*a* = 1.0169	*b* = 0.0000	0.9869	0.3096	0.0086	0.0686	0.0926	−30.0767	−16.7434

Model coefficients (*a*, *b*, *c*, *k*, and *n*), *R*^2^ (coefficient of determination), *χ*^2^ (chi-squared statistic), *MSE* (mean squared error), *SSE* (sum of square error), *RMSE* (root mean square error), *AIC* (Akaike information criterion), and *AIC*_c_ (corrected Akaike information criterion).

**Table 4:** Proximate composition of dietary fiber components of DRP at different drying temperatures.

Treatments	TDF (g∙100 g^−1^ db)	IDF (g∙100 g^−1^ db)	SDF (g∙100 g^−1^ db)	SDF:IDF
Freeze-drying	64.14 ± 1.06 ^a^	61.78 ± 0.91 ^ab^	2.35 ± 0.15 ^b^	0.04:1
50 °C	64.29 ± 3.56 ^a^	61.51 ± 3.23 ^a^	2.77 ± 0.36 ^ab^	0.05:1
60 °C	64.63 ± 2.99 ^a^	61.54 ± 2.53 ^a^	3.08 ± 0.47 ^ab^	0.05:1
70 °C	59.40 ± 3.11 ^a^	56.03 ± 3.12 ^b^	3.37 ± 0.19 ^a^	0.06:1
80 °C	61.43 ± 1.36 ^a^	58.55 ± 1.18 ^b^	3.87 ± 0.43 ^a^	0.07:1
90 °C	61.94 ± 1.52 ^a^	58.53 ± 1.41 ^b^	3.40 ± 0.16 ^a^	0.06:1

Values are presented as mean ± SD of triplicate experiments. Means with different letters (a, b) within each column indicate significant differences (Tukey test, *p* < 0.05). IDF (insoluble dietary fiber), SDF (soluble dietary fiber), TDF (total dietary fiber), and db (dry basis).

**Table 5:** Techno-functional attributes of DRP subjected to different drying temperatures.

Treatments	SOL (%)	WHC (mL∙g^−1^ db)	OHC (mL∙g^−1^ db)	SC (mL∙g^−1^ db)	TD (kg∙m^−3^ db)
Freeze-drying	76.0 ± 0.1 ^a^	11.8 ± 0.6 ^a^	3.3 ± 0.3 ^a^	15.2 ± 0.3 ^a^	279.0 ± 4.7 ^c^
50 °C	65.2 ± 1.1 ^c^	8.0 ± 0.2 ^b^	2.4 ± 0.2 ^b^	7.6 ± 0.3 ^b^	313.9 ± 5.9 ^bc^
60 °C	68.8 ± 2.1 ^b^	8.3 ± 0.4 ^b^	2.3 ± 0.1 ^b^	8.1 ± 0.4 ^b^	311.5 ± 8.2 ^bc^
70 °C	71.8 ± 0.8 ^ab^	8.2 ± 0.5 ^b^	3.0 ± 0.3 ^a^	6.9 ± 0.3 ^c^	314.3 ± 8.3 ^b^
80 °C	72.5 ± 0.9 ^a^	7.7 ± 0.2 ^bc^	1.7 ± 0.2 ^c^	7.0 ± 0.2 ^c^	322.6 ± 19.7 ^b^
90 °C	72.6 ± 1.8 ^a^	7.0 ± 0.5 ^c^	2.0 ± 0.1 ^c^	7.1 ± 0.0 ^c^	362.1 ± 24.5 ^a^

Values are presented as the mean ± SD of triplicate experiments. Means with different letters (a–c) within each column indicate significant differences (Tukey test, *p* < 0.05). SOL (solubility), WHC (water-holding capacity), OHC (oil-holding capacity), SC (swelling capacity), TD (tapped density), and db (dry basis).

## Data Availability

The data generated in this work are available upon request from the corresponding author. The data are not publicly available due to privacy restrictions.

## Supplementary Materials

The following supporting information can be downloaded at: https://www.mdpi.com/article/10.3390/foods13223597/s1. Table S1: Physicochemical characterization of raspberries (*Rubus idaeus*, Var. ‘Adelita’). Table S2: Linear regression equations derived from ln *MR* versus drying time for different drying temperatures. Figure S1: Plot of the natural logarithm of effective moisture diffusivity (ln *D*_eff_) versus the inverse of absolute temperature (1∙T^−1^) for raspberry pomace dried at different temperatures (50, 60, 70, 80, and 90 °C). Figure S2: Linear regression of the natural logarithm of the moisture ratio (ln *MR*) versus drying time for raspberry pomace at different drying temperatures (50, 60, 70, 80, and 90 °C).
