# Supplementary material for: Process Modeling and Convective Drying Optimization of Raspberry Pomace as a Fiber-Rich Functional Ingredient: Effect on Techno-Functional and Bioactive Properties
Source: Foods. 2024 Nov 11;13(22):3597. doi: 10.3390/foods13223597 (PMC11594013; doi:10.3390/foods13223597)
Supplement: Supplementary file 1 [file foods-13-03597-s001.zip › foods-3280673-supplementary.pdf]

Process Modeling and Convective Drying Optimization of Raspberry Pomace as a Fiber-Rich Functional Ingredient: Effect on Techno-functional and bioactive Properties

José P. Tejeda-Miramontes[1], Brenda C. Espinoza-Paredes[1], Ana Zatarain-Palffy[1], Tomás García-Cayuela[1], Viridiana Tejada-Ortigoza[2], Luis E. Garcia-Amezquita[2]*

[1]Tecnologico de Monterrey, Escuela de Ingenieria y Ciencias, Campus Guadalajara, Ave. General Ramón Corona 2514, Zapopan, 45138, México.

[2]Tecnologico de Monterrey, Escuela de Ingenieria y Ciencias, Campus Monterrey, Ave. Eugenio Garza Sada 2501, Monterrey, 64849, México.

*Corresponding author: garcia.amezquita@tec.mx (L.E.G.A.)

Table S1. Physicochemical characterization of raspberries (*Rubus idaeus*, Var. 'Adelita').

Parameter	Value
pH	3.50 ± 0.01
Soluble solids (°Bx)	11.2 ± 0.15
Titratable acidity [a]	0.75 ± 0.13%
Maturity index [b]	14.05 ± 0.25
Color	
*L**	39.12 ± 0.15
*a**	34.59 ± 0.41
*b**	13.74 ± 0.24

Mean values represent the average ± SD of triplicate experiments. Color parameters (*L**, *a**, *b**) follow the CIELAB color space, where *L** indicates lightness, *a** red/green axis, and *b** yellow/blue axis.

[a] Titratable acidity expressed as g of citric acid per 100 g of juice.

[b] °Bx/acidity.

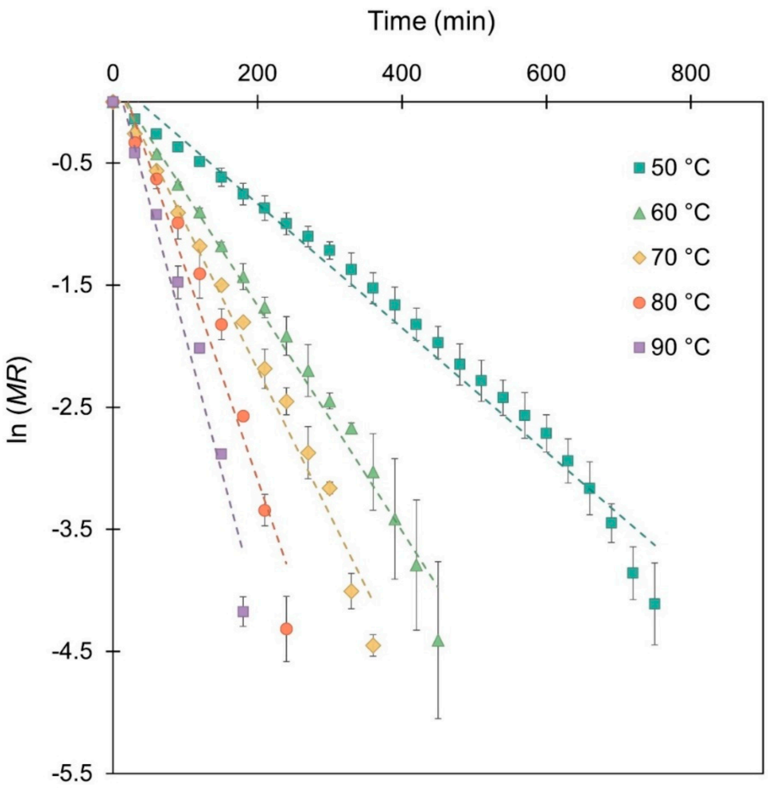

Figure S1. Linear regression of the natural logarithm of the moisture ratio (ln MR) versus drying time for raspberry pomace at different drying temperatures (50, 60, 70, 80, and 90 °C).

[a]The slopes of these lines were used to calculate the effective moisture diffusivity (D_{eff}).

[b]Error bars represent the standard deviation of triplicate measurements.

Table S2. Linear regression equations derived from ln MR versus drying time for different drying temperatures.

Temperature (°C)	Equation
50	Y= -0.0051x + 0.1821
60	Y= -0.0092x + 0.1736
70	Y= -0.0119x + 0.1916
80	Y= -0.0172x + 0.3548
90	Y= - 0.0221x + 0.2881

Linear regression equations obtained from the drying data of raspberry pomace in **Figure S1**. These equations were used to calculate the effective moisture diffusivity (D_{eff}) for each temperature.

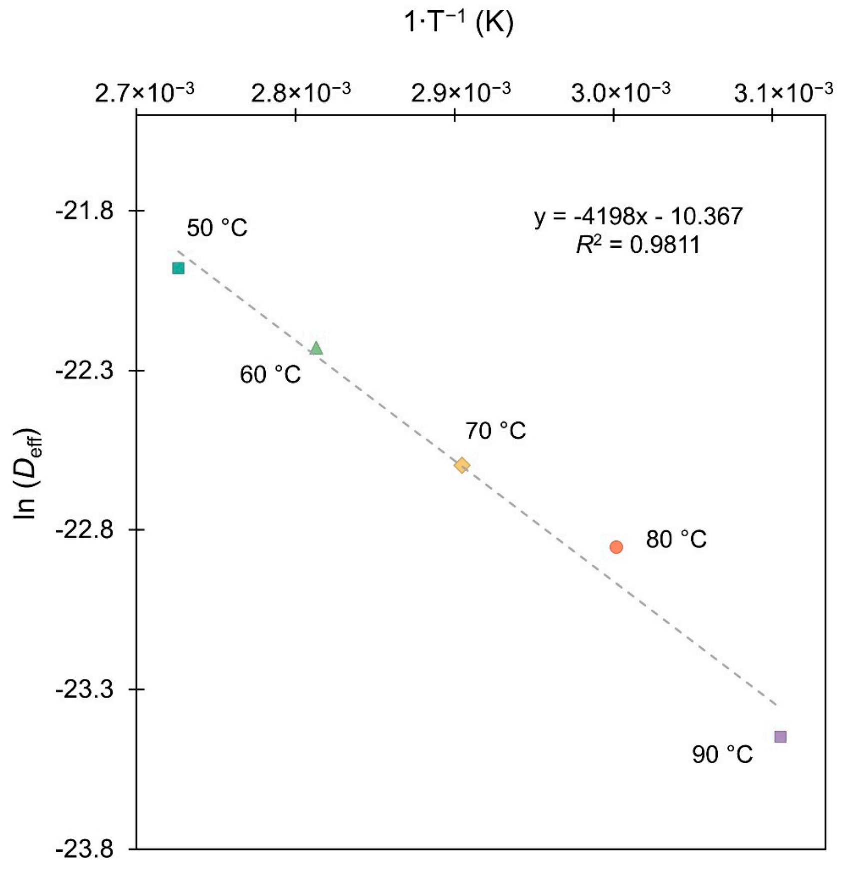

Figure S2. Plot of the natural logarithm of effective moisture diffusivity (ln D_{eff}) versus the inverse of absolute temperature ($1{\cdot}T^{-1}$) for raspberry pomace dried at different temperatures (50, 60, 70, 80, and 90 °C).

[a]The slope of the linear regression was used to calculate the activation energy (E_a) according to the Arrhenius equation.

[b]The regression equation and R^2 value are provided, showing the quality of the fit.
